# The association of prenatal and postnatal macrolide exposure with subsequent development of infantile hypertrophic pyloric stenosis: a systematic review and meta-analysis

**DOI:** 10.1186/s13052-019-0613-2

**Published:** 2019-02-04

**Authors:** Hamdi H. Almaramhy, Abdulmohsen H. Al-Zalabani

**Affiliations:** 1 Department of Surgery (Pediatric Surgery Division), College of Medicine, Taibah University, Madinah, Saudi Arabia; 2 Department of Family and Community Medicine, College of Medicine, Taibah University, PO box 42317, Madinah, 41541 Saudi Arabia

**Keywords:** Pyloric stenosis, Macrolide, Erythromycin, Congenital defect, Infant

## Abstract

**Background:**

The association between macrolides use and subsequent occurrence of infantile hypertrophic pyloric stenosis (IHPS) is still debatable. The aim of this study was to conduct a systematic review and meta-analysis of the association between perinatal exposure to macrolides, mainly erythromycin, and the development of pyloric stenosis.

**Methods:**

Original studies were identified using MEDLINE, Web of Science, Scopus, Google Scholar, and the Cochrane Library databases. Studies investigating the association between perinatal exposure to macrolides and pyloric stenosis were included. The most adjusted effect estimates were pooled using random-effects meta-analysis. The I^2^ and Egger’s tests were used to assess heterogeneity and publication bias, respectively.

**Results:**

Fourteen papers (12 retrospective cohort studies and two case-control studies) were included. For postnatal exposure, the overall estimate of seven cohort studies indicated a statistically significant association (RR = 3.17, 95% CI: 2.38–4.23; I^2^ = 10.0%) with no evidence of publication bias (Egger *P* = 0.81). For prenatal exposure, six cohort studies and two case-control studies were included. Meta-analysis demonstrated a statistically significant association in the cohort studies (OR = 1.47, 95% CI: 1.03–2.09; I^2^ = 29.3%), but not in the case-control studies (OR = 1.02, 95% CI: 0.66–1.58; I^2^ = 51.2%). The overall pooled result was not statistically significant. Only two studies were included for exposure through breastfeeding, and the estimates did not show a statistically significant association (OR = 1.31; 95% CI: 0.42–4.1; I^2^ = 69.1%).

**Conclusions:**

The study demonstrated good evidence of association between development of IHPS and direct postnatal exposure to macrolides. However, the evidence on the effects of prenatal exposure or postnatal maternal exposure (breastfeeding) is not conclusive.

## Background

Infantile hypertrophic pyloric stenosis (IHPS) is a common cause of gastrointestinal obstruction in infancy, affecting up to three of 1000 live births [1, 2]. It is also the most acquired cause for surgical operation in the early days of life [3]. The underlying pathology is muscle hypertrophy at the pyloric region [4], which leads to obstruction of the gastric outlet. The infant commonly presents with projectile non-bilious vomiting and some degree of dehydration in the first 2–12 weeks of life. Initially, investigators thought that IHPS had a congenital origin, but it is now believed to be an acquired condition. The exact etiology remains unknown, but genetic and environmental factors have been implicated as risk factors for IHPS occurrence [5–8]. For example, having a caesarean section, prematurity, primiparity, young maternal age, and smoking were reported as significant IHPS risk factors [9]. IHPS has also been shown to have strong familial aggregation, especially among twins [10].

One suspected environmental risk factor is the exposure to macrolides—mainly erythromycin—in early infancy; however, supporting evidence for this association remains elusive [11]. Both infants and mothers could need antibiotics during the perinatal period, including macrolides. For example, macrolides are the recommended medications for treatment and prophylaxis of pertussis in infants [12], and erythromycin is recommended for the treatment of chlamydia infection in pregnancy. The proposed explanation of the association with pyloric stenosis is that erythromycin, which is a motilin agonist, interacts with motilin receptors and this stimulates contraction of the gastrointestinal smooth muscles; these contractions could produce hypertrophy of the pylorus [13]. The aim of the present study was to conduct a systematic review and meta-analysis of the association between exposure to macrolides, including erythromycin, and the development of pyloric stenosis.

## Methods

### Identification of studies

A systematic literature search was conducted to identify articles that discussed the association between macrolide exposure and the development of pyloric stenosis by accessing the following databases: MEDLINE (PubMed interface), Web of Science, Scopus (including EMBASE records), Google Scholar, and the Cochrane Library. The search was carried out until May 2018 and had no time or language restrictions. The following terms were used both as medical subject heading terms and keywords in the search process: pyloric stenosis, pyloric hypertrophy, pyloric stricture, pyloromyotomy, and macrolides as well as individual macrolide antibiotic names (e.g. erythromycin). The full search strategy is available in Appendix A. References in the selected articles were further explored to find other relevant studies for the analysis. Data retrieval was performed independently by the two investigators and further cross-checked. EndNote software was used to compile references and to check and remove duplicates.

### Study selection

The articles identified in the literature search process were selected based on the title and abstract in the first stage, and then based on the full text of selected articles in the second stage. We included articles that satisfied the following criteria: i) investigated the association between infant exposure to macrolides (including infant use, as well as prenatal and postnatal maternal use) and pyloric stenosis; ii) reported original data from interventional or observational study; and iii) reported effect measures with their confidence intervals or data required to calculate them. The exposure of interest was infant exposure to macrolides, including prenatal and postnatal exposure, while the outcome of interest was pyloric stenosis. The selection was performed by one researcher and double-checked independently by the other researcher. The most adjusted effect estimate from each included study was used for the meta-analysis.

### Data extraction

Relevant data extracted from the selected articles included the first author, country, study design, study period, ascertainment of exposure and outcome, data analysis methods, exposure categories and timing, crude and adjusted effect estimates, and confounding factors used for adjustment.

### Assessment of risk of bias

We assessed the risk of bias in individual studies using the Newcastle-Ottawa Scale for assessing the quality of nonrandomized studies in meta-analyses [14]. This scale uses a star system to assess the risk of bias, where each study is given a number of stars out of a total of nine possible stars. When no explicit statement specified what factors were adjusted in the analysis, the analysis was considered not adjusted.

### Statistical analysis

The primary meta-analysis was based on random-effects modeling to calculate the overall odds ratio (OR) and 95% confidence interval (CI) for pyloric stenosis to compare macrolide exposure versus non-exposure. For meta-analysis, we selected the most adjusted estimate from each study. We used the OR or the risk ratio reported by the original studies; however, if the rate ratio was reported, we used the raw data to calculate OR for use in the meta-analysis. Two studies reported results with zero cells in one of the arms. In those cases, we used the method of adding 0.5 to each cell [15]. However, if a study had zero cells in both arms, we discarded it from meta-analysis.

We executed separate meta-analyses for direct infant exposure, prenatal maternal exposure, and postnatal maternal exposure. The I^2^ test statistic was used to assess the heterogeneity of the studies. Egger’s linear regression method was used to assess publication bias. Stata 13 was used for all statistical analyses. We followed the Preferred Reporting Items for Systematic Reviews and Meta-Analyses (PRISMA) [16] guidelines and Meta-analysis of Observational Studies in Epidemiology (MOOSE) [17] to report the results. This study was exempted from review by the College of Medicine Institutional Review Board.

## Results

### Description of studies

**Fig. 1:**
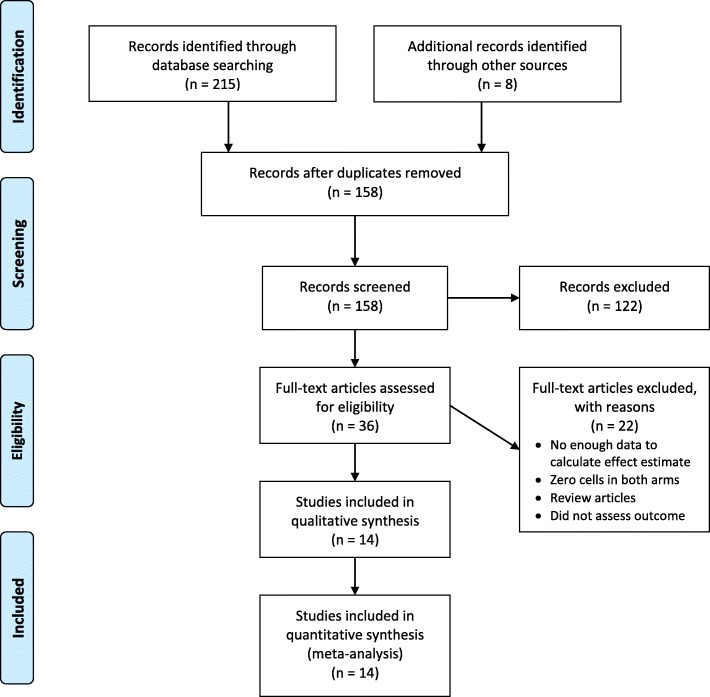
PRISMA flow diagram outlining study selection

In total, 14 eligible articles were identified that involved a total 7755 cases of pyloric stenosis. Figure 1 summarizes the selection process. The selected articles consisted of 12 retrospective cohort studies [2, 4, 18–27] and two case-control studies [28, 29]. Two further randomized controlled trials [30, 31] were also identified but were excluded because they had no events (zero cells) in both arms. Table 1 summarizes the studies included in the current systematic review.

**Table 1:** Characteristics of the included studies^a^

Author, year	Study design	Country	Study Interval	Cases/subjects	Exposure	Adjusted risk estimate (95% CI)	Quality	Adjustment variables
Infant exposure								
Eberly,2015 [21]	R-COH	USA	2001–2012	2466/1074236	**Erythromycin:** 0–14 days 15–42 days 43–90 days 0–90 days **Azithromycin:** 0–14 days 15–42 days 0–90 days	13.3 (6.8–25.9) 4.1 (1.69–9.91) 1.19 (0.38–3.71) 3.94 (2.44–6.36) 8.26 (2.62–26) 2.98 (1.24–7.2) 0.71 (0.36–1.43)	9	firstborn status, gender, and the year of the birth
Ericson,2015 [22]	R-COH	USA	1997–2012	86/20196	Metoclopramide vs Erythromycin (0–120 days)	0.52 (0.26–1.02)	9	Gestational age at birth, small-for-gestational-age status, severity of illness, and age at first medication exposure
Lund,2014 [26]	R-COH	Denmark	1996–2011	849/998529	**Erythromycin:** 0–13 days 14–120 days	Rate Ratio: 29.8 (16.4–54.1) 3.24 (1.2–8.74)	9	birth order, gender, calendar period, current age of the infant, gestational age at birth, small for gestational age status, caesarean section, major congenital malformations, and maternal smoking during pregnancy
Cooper, 2002 [19]	R-COH	USA	1985–1997	804/314029	**Erythromycin:** 3–13 days 14–27 days 28–90 days Any use	incidence rate ratio: 7.88 (1.97–31.57) 0.92 (0.13–6.57) 1.95 (0.87–4.38) 2.05 (1.06–3.97)	9	Child’s age, sex, and race
Mahon, 2001 [27]	R-COH	USA	1993–1999	43/14876	**Erythromycin:** ≤1 week ≤2 week ≤3 months	10.62 (4.2–26.7) 10.51 (4.5–24.7) 4.98 (2.1–11.7)	7	NR
Honein,1999 [23]	R-COH	USA	Jan - Feb, 1999	7/282	**Erythromycin**	∞ (1·7–∞)	6	Not adjusted
Ludvigsson, 2016 [4]	R-COH	Sweden	2005–2010	450/582494	**Macrolides** (0–120 days)	NR	6	NR
Maternal use during pregnancy								
Mahon, 2001 [27]	R-COH	USA	1993–1999	43/14876	**Erythromycin:** Any time during pregnancy	1.19 (0.6–2.3)	7	NR
Cooper, 2002 [20]	R-COH	USA	1985–1997	679/260799	**Erythromycin:** Any time during pregnancy ≥ 32 weeks **Other macrolides:** Any time during pregnancy ≥ 32 weeks	1.15 (0.84–1.56) 1.17 (0.84–1.64) 2.77 (1.22–6.30) 2.45 (0.78–7.68)	9	sex, race, first-born status, year of birth, and infant’s postnatal prescriptions for erythromycin
Källén, 2005 [24]	R-COH	Sweden	1995–2002	464/677028	**Erythromycin:** Any time during pregnancy 1st trimester	2.51 (0.92–5.46) 3.03 (1.08–8.50)	7	NR
Rookkapan, 2008 [25]	R-COH	Denmark	1991–2005	NR/176905	**Erythromycin:** Any time during pregnancy	1.05 (0.43–2.55)	7	NR
Dinur, 2013 [18]	R-COH	Israel	1999–2009	50/102831	**Macrolides:** 3rd trimester	NR	7	Not adjusted
Lund,2014 [26]	R-COH	Denmark	1996–2011	877/999378	**Macrolides:** 0–27 weeks ≥28 weeks	1.02 (0.65–1.59) 1.77 (0.95–3.31)	9	birth order, sex, calendar period, and current age of the infant
Louik, 2002 [29]	C-C	USA	1976–1998	1044/1704	**Erythromycin:** 1–24 weeks 25–40 weeks 33–40 weeks	1.0 (0.6–1.6) 0.6 (0.3–1.1) 0.7 (0.3–1.8)	8	maternal age, geographical region, study period, parity, sex of infants, gestational age
Lin,2013 [28]	C-C	USA/Canada	1994 to 2008	735/6952	**Erythromycin:** 1st trimester 2nd trimester 3rd trimester **Macrolides:** 1st trimester 2nd trimester 3rd trimester	0.9 (0.3–3.0) 1.5 (0.4–4.8) 1.5 (0.5–5.1) 1.3 (0.6–2.8) 1.3 (0.5–3.0) 1.3 (0.6–2.9)	8	residence and year, maternal age, race, education, pre-pregnancy BMI, family history of congenital malformations, diabetes mellitus, first trimester cigarette smoking, peri-conceptional folic acid supplement, multiple pregnancy, infections, sexually transmitted disease, febrile events
Maternal use after birth								
Lund,2014 [26]	R-COH	Denmark	1996–2011	849/999378	**Macrolides:** 0–13 days 14–120 days	Rate Ratio: 3.49 (1.92–6.34) 0.7 (0.26–1.9)	9	birth order, sex, calendar period, and current age of the infant, age at birth, small for gestational age, caesarean section, major congenital malformations, and maternal smoking during pregnancy
SØRENSEN, 2003 [2]	R-COH	Denmark	1991–2000	78/35856	**Macrolides:** (0–42 days)	2.8 (0.7–11.5)	9	maternal age, birth order and smoking status

^a^ Abbreviations: R-COH: Retrospective cohort studies; C-C: case-control studies; CI: Confidence Interval; NR: Not Reported

### Infant exposure (postnatal direct use)

**Fig. 2:**
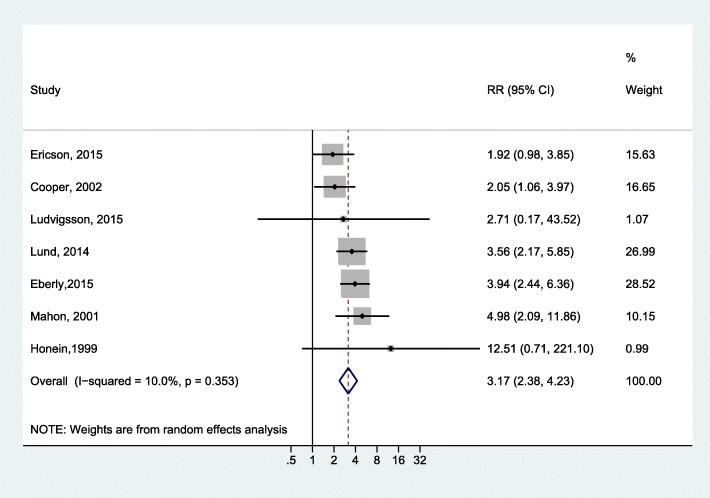
Forest plot for the meta-analysis of the estimates for the association between infant exposure to erythromycin and IHPS

We investigated the association between infant exposure (up to 120 days of life) to erythromycin and IHPS by pooling the results of seven cohort studies [4, 19, 21–23, 26, 27]. The overall estimate indicated a statistically significant association (RR = 3.17, 95% CI: 2.38–4.23; I^2^ = 10.0%; Fig. 2). We found no evidence of publication bias (Egger *P* = 0.81).

We noticed potentially inconsistent results in the study by Cooper et al. [19], who reported an adjusted incident rate ratio of 2.05 (95% CI: 1.06–3.97). However, calculating the OR from the reported raw data gave an OR of 0.49 (95% C.I. = 0.25–0.94), which was the reciprocal of the ratio and 95% C.I. reported in the paper. We contacted the corresponding author, who explained that the reported result was the adjusted estimate. Our main analysis discussed above was based on the reported adjusted estimate. However, we tested the effect of this study on the meta-analysis results by running another meta-analysis using estimates based on the raw data from the Cooper study; the analysis still showed a positive association (RR = 2.52, 95% CI: 1.24–5.15), but with a high level of heterogeneity among studies (I^2^ = 81.7%). In addition, running the meta-analysis while omitting the Cooper study produced similar results to the estimates obtained by our main analysis (RR = 3.47; 95% CI: 2.60–4.62; I^2^ = 0.0%).

Four studies provided separate reports on the association between the exposure to erythromycin during first two weeks of life and IHPS. All four studies found a positive association and had a higher effect estimate compared to those reported by the same studies for anytime exposure. Specifically, Mahon et al. [27] reported a relative risk of 10.51 (95% CI, 4.5–24.7) and Eberly et al. [21] reported an OR of 13.3 (95% CI, 6.8–25.9), whereas Cooper et al. [19] reported an incidence rate ratio of 7.88 (95% CI, 1.97–31.57) and Lund et al. [26] reported a rate ratio of 29.8 (95% CI, 16.4–54.1). The effect estimates for erythromycin exposure during first two weeks of life could not be pooled due to the varying types of effect estimates used in the four individual studies.

### Prenatal exposure (maternal use during pregnancy)

**Fig. 3:**
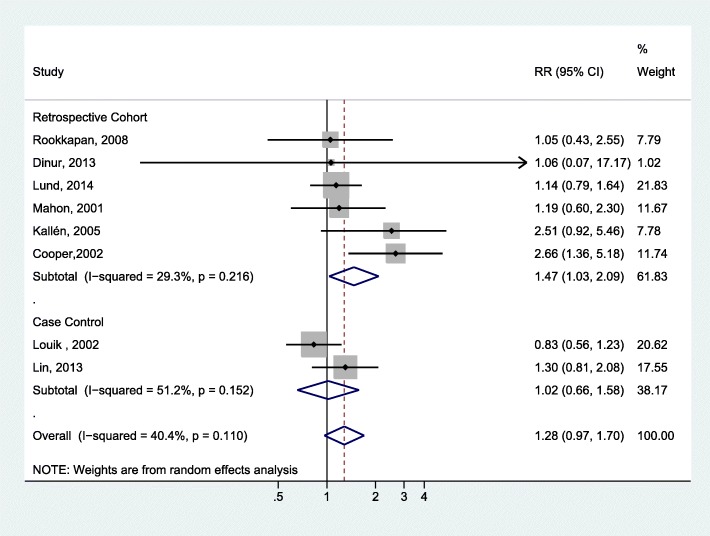
Forest plot for the meta-analysis of the estimates for the association between IHPS and prenatal exposure to macrolide by study design

Six cohort studies [18, 20, 24–27] and two case-control studies [28, 29] reported on the association between prenatal exposure to macrolides and IHPS. Meta-analysis of these studies (Fig. 3) demonstrated a statistically significant association in the cohort studies (OR = 1.47, 95% CI: 1.03–2.09; I^2^ = 29.3%), but not in the case-control studies (OR = 1.02, 95% CI: 0.66–1.58; I^2^ = 51.2%). The overall pooled results did not achieve statistical significance (OR = 1.28, 95% CI: 0.97–1.70; I^2^ = 40.4%) and no publication bias was evident (Egger *P* = 0.309).

**Fig. 4:**
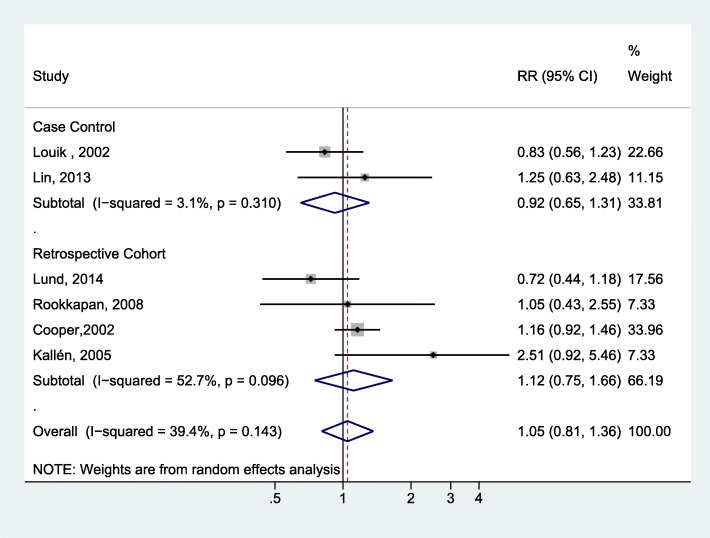
Forest plot for the meta-analysis of the estimates for the association between IHPS and prenatal exposure to erythromycin by study design

We separately assessed the association with prenatal exposure to erythromycin by pooling the estimates from four cohort studies [20, 24–26] and two case-control studies [28, 29] (Fig. 4). No statistically significant association was noted among the cohort studies (OR = 1.12; 95% CI: 0.75–1.66; I^2^ = 52.7%) or the case-control studies (OR = 0.92; 95% CI: 0.65–1.31; I^2^ = 3.1%).

### Postnatal maternal exposure (indirect infant exposure through lactation)

Only two studies [2, 26] were identified that assessed the association between IHPS and postnatal maternal exposure while breastfeeding the infant. Meta-analysis of the estimates from these two studies did not show any statistically significant association (OR = 1.31; 95% CI: 0.42–4.1; I^2^ = 69.1%).

## Discussion

This meta-analysis study reviewed the association between macrolide exposure, focusing on erythromycin, and the development of pyloric stenosis in infants at different times of exposure: prenatal, direct postnatal infant use, and indirect postnatal through maternal use and feeding.

The main finding in this meta-analysis was the strong association between direct postnatal infant exposure to erythromycin and IHPS (RR = 3.17, 95% CI: 2.38–4.23). Individual studies reporting on the time of exposure have indicated a strong association for early exposure during the first two weeks of life. Conversely, indirect exposure through maternal use, either prenatally or postnatally, has a limited association with the risk of IHPS.

For prenatal exposure, our meta-analysis of cohort studies showed a modest increase in risk with marginal statistical significance for prenatal exposure to macrolides; however, the pooled results of the cohort and case-control studies could not achieve statistical significance. Prenatal erythromycin exposure was not associated with IHPS based on the pooled results of either the cohort studies or the case-control studies. Although the placental transfer of erythromycin is documented, the amount transferred is limited to around 2.6% of the maternal serum concentration [32]. Limited transplacental transfer has also been observed for roxithromycin and azithromycin [33]. In contrast, clarithromycin has shown a high transplacental transfer [34]. Different macrolides appear to have different transplacental transfer capacities, which may explain the mixed results among individual studies and the less prominent effect of erythromycin exposure during pregnancy compared to direct postnatal infant exposure.

For postnatal maternal exposure (breastfeeding), our pooled results did not show a statistically significant association with IHPS. Biologically, the macrolides are secreted in breast milk; however, the measurable levels in breast milk are much smaller than the standard therapeutic doses used in infants [26]. Noticeably, Lund et al. reported a higher rate ratio among infants exposed to macrolides through breastfeeding during the first two weeks of life (Rate Ratio = 3.49; 95% CI = 1.92–6.34) compared to infants exposed during the 14–120 days period (Rate Ratio = 0.7; 95% CI = 0.26–1.90) [26]. This finding agrees with those for direct infant exposure discussed above, where exposure during the first two weeks of life had a higher risk of IHPS.

A common theory for explaining how erythromycin can lead to IHPS is its interaction with motilin receptors [23]. Erythromycin is proposed to act as a motilin agonist that leads to increased activity of the migrating motor complexes and strong gastric and duodenal contractions. Frequent contractions, in turn, are believed to lead to pyloric hypertrophy and subsequent obstruction. This theory is supported by the delayed occurrence of IHPS among premature infants, since motilin receptors are not functional prior to 32 weeks of gestation [23]. In fact, Hussain and Herson [35] conducted a preliminary database analysis and concluded that premature infants maybe less susceptible to IHPS as a result of exposure to erythromycin compared to term infants.

The present study has also some limitations. First, most of the included studies are based on registry data. Although the use of registry data overcomes the problem of recall bias, the ascertainment of exposure and outcome is not always optimum. For example, registry data can confirm a medication prescription, but cannot guarantee adherence to the antibiotic protocol by the women or infants (except for hospitalized patients). Moreover, the completeness of the data within the registry databases was also questioned previously due to the delayed registration of infant data [36]. Second, the number of studies included in the analysis was small, especially when we studied the association with postnatal maternal use. Only two studies have been identified regarding the association between IHPS and postnatal maternal exposure (breastfeeding). Although we followed the recommended practice [37, 38] and opted to pool the results to get the best available estimate, the results need cautious interpretation due to the limited number of underlying studies. Finally, the accuracy of the risk estimate may be influenced by the quality of the included studies. We assessed the risk of bias in individual studies using a recommended tool (NOS); two studies scored six out of nine, while the remaining studies scored seven or higher.

## Conclusion

This meta-analysis synthesizes the best available evidence to give a single estimate of the association between perinatal macrolide exposure and IHPS, providing valuable information for researchers, clinicians, and policy-makers. In summary, good evidence supports an increased risk of IHPS due to direct infant exposure to erythromycin. However, the evidence on the effects of prenatal or postnatal maternal exposure is not conclusive and the current research available does not support the presence of an association.

## Appendix A: PubMed search strategy

1. Macrolides OR azithromycin OR clarithromycin OR erythromycin OR telithromycin OR spiramycin OR midecamycin OR oleandomycin OR Fidaxomicin OR roxithromycin OR josamycin OR troleandomycin OR miocamycin OR dirithromycin OR flurithromycin
2. ((Pyloric stenosis) OR (pyloric hypertrophy) OR pylorostenosis OR (gastric outlet obstruction) OR (pyloric stricture) OR (hypertrophied pylorus) OR pyloromyotomy OR (intestinal obstruction))
3. #1 AND #2

## References

[1] de Laffolie J, Turial S, Heckmann M, Zimmer K-P, Schier F (2012). Decline in infantile hypertrophic pyloric stenosis in Germany in 2000–2008. Pediatrics.

[2] Sørensen HT, Skriver MV, Pedersen L, Larsen H, Ebbesen F, Schonheyder HC (2003). Risk of infantile hypertrophic pyloric stenosis after maternal postnatal use of macrolides. Scand J Infect Dis.

[3] Georgoula C, Gardiner M (2012). Pyloric stenosis a 100 years after Ramstedt. Arch Dis Child.

[4] Ludvigsson JF, Lundholm C, Ortqvist AK, Almqvist C (2016). No association between macrolide treatment in infancy and later pyloric stenosis in Sweden. Eur J Epidemiol.

[5] Chung E (2008). Infantile hypertrophic pyloric stenosis: genes and environment. Arch Dis Child.

[6] Everett KV, Chioza BA, Georgoula C, Reece A, Gardiner RM, Chung EM (2009). Infantile hypertrophic pyloric stenosis: evaluation of three positional candidate genes, TRPC1, TRPC5 and TRPC6, by association analysis and re-sequencing. Hum Genet.

[7] Stevenson RE, Hall JG, Everman DB, Solomon BD (2016). Human malformations and related anomalies.

[8] Hu M, Craig J, Howard N, Kan A, Chaitow J, Little D (2004). A novel mutation of WT1 exon 9 in a patient with Denys-Drash syndrome and pyloric stenosis. Pediatr Nephrol.

[9] Svenningsson A, Svensson T, Akre O, Nordenskjold A (2014). Maternal and pregnancy characteristics and risk of infantile hypertrophic pyloric stenosis. J Pediatr Surg.

[10] Krogh C, Fischer TK, Skotte L, Biggar RJ, Oyen N, Skytthe A (2010). Familial aggregation and heritability of pyloric stenosis. JAMA.

[11] Ranells JD, Carver JD, Kirby RS (2011). Infantile hypertrophic pyloric stenosis: epidemiology, genetics, and clinical update. Adv Pediatr Infect Dis.

[12] Tiwari T, Murphy TV, Moran J, National Immunization Program CDC (2005). Recommended antimicrobial agents for the treatment and postexposure prophylaxis of pertussis: 2005 CDC guidelines. MMWR Recomm Rep.

[13] Everett KV, Chioza BA, Georgoula C, Reece A, Capon F, Parker KA (2008). Genome-wide high-density SNP-based linkage analysis of infantile hypertrophic pyloric stenosis identifies loci on chromosomes 11q14-q22 and Xq23. Am J Hum Genet.

[14] Wells G, Shea B, O'Connell D, Peterson J, Welch V, Losos M, et al. The Newcastle-Ottawa Scale (NOS) for assessing the quality of nonrandomised studies in meta-analyses: Ottawa Hospital Research Institute; 2014. Available from: http://www.ohri.ca/programs/clinical_epidemiology/oxford.asp. [updated 2014; cited 10 Mar 2018]

[15] Egger M, Smith GD, Altman DG (2001). Systematic reviews in health care: meta-analysis in context.

[16] Moher D, Liberati A, Tetzlaff J, Altman DG, Group P (2009). Preferred reporting items for systematic reviews and meta-analyses: the PRISMA statement. PLoS Med.

[17] Stroup DF, Berlin JA, Morton SC, Olkin I, Williamson GD, Rennie D (2000). Meta-analysis of observational studies in epidemiology: a proposal for reporting. Meta-analysis of observational studies in epidemiology (MOOSE) group. JAMA.

[18] Bahat Dinur A, Koren G, Matok I, Wiznitzer A, Uziel E, Gorodischer R (2013). Fetal safety of macrolides. Antimicrob Agents Chemother.

[19] Cooper WO, Griffin MR, Arbogast P, Hickson GB, Gautam S, Ray WA (2002). Very early exposure to erythromycin and infantile hypertrophic pyloric stenosis. Arch Pediatr Adolesc Med.

[20] Cooper WO, Ray WA, Griffin MR (2002). Prenatal prescription of macrolide antibiotics and infantile hypertrophic pyloric stenosis. Obstet Gynecol.

[21] Eberly MD, Eide MB, Thompson JL, Nylund CM (2015). Azithromycin in early infancy and pyloric stenosis. Pediatrics.

[22] Ericson JE, Arnold C, Cheeseman J, Cho J, Kaneko S, Wilson E (2015). Use and safety of erythromycin and metoclopramide in hospitalized infants. J Pediatr Gastroenterol Nutr.

[23] Honein MA, Paulozzi LJ, Himelright IM, Lee B, Cragan JD, Patterson L (1999). Infantile hypertrophic pyloric stenosis after pertussis prophylaxis with erythromcyin: a case review and cohort study. Lancet.

[24] Kallen BA, Otterblad Olausson P, Danielsson BR (2005). Is erythromycin therapy teratogenic in humans?. Reprod Toxicol.

[25] Rookkapan K, Nørgaard M, Wogelius P, Gislum M, Mahon BE, Sørensen HT (2008). Maternal use of erythromycin and risk of congenital malformation and infantile hypertrophic pyloric stenosis: a Danish population-based cohort study. Pharmacoepidemiol Drug Saf.

[26] Lund M, Pasternak B, Davidsen RB, Feenstra B, Krogh C, Diaz LJ (2014). Use of macrolides in mother and child and risk of infantile hypertrophic pyloric stenosis: nationwide cohort study. BMJ.

[27] Mahon BE, Rosenman MB, Kleiman MB (2001). Maternal and infant use of erythromycin and other macrolide antibiotics as risk factors for infantile hypertrophic pyloric stenosis. J Pediatr.

[28] Lin KJ, Mitchell AA, Yau WP, Louik C, Hernandez-Diaz S. Safety of macrolides during pregnancy. Am J Obstet Gynecol 2013;208(3):221 e1–8. 10.1016/j.ajog.2012.12.023 PMC3581717 23254249

[29] Louik C, Werler MM, Mitchell AA (2002). Erythromycin use during pregnancy in relation to pyloric stenosis. Am J Obstet Gynecol.

[30] Mohammadizadeh M, Ghazinour M, Iranpour R (2010). Efficacy of prophylactic oral erythromycin to improve enteral feeding tolerance in preterm infants: a randomised controlled study. Singap Med J.

[31] Ng PC, So KW, Fung KS, Lee CH, Fok TF, Wong E (2001). Randomised controlled study of oral erythromycin for treatment of gastrointestinal dysmotility in preterm infants. Arch Dis Child Fetal Neonatal Ed.

[32] Bulska M, Szczesniak P, Pieta-Dolinska A, Oszukowski P, Orszulak-Michalak D (2015). The placental transfer of erythromycin in human pregnancies with group B streptococcal infection. Ginekol Pol.

[33] Heikkinen T, Laine K, Neuvonen PJ, Ekblad U (2000). The transplacental transfer of the macrolide antibiotics erythromycin, roxithromycin and azithromycin. BJOG.

[34] Witt A, Sommer EM, Cichna M, Postlbauer K, Widhalm A, Gregor H (2003). Placental passage of clarithromycin surpasses other macrolide antibiotics. Am J Obstet Gynecol.

[35] Hussain N, Herson VC (2002). Erythromycin use during pregnancy in relation to pyloric stenosis. Am J Obstet Gynecol.

[36] de Vries F (2014). The difficulty in evaluating all findings in study on use of macrolides in mother and child and risk of infantile hypertrophic pyloric stenosis. BMJ.

[37] Borenstein M. Introduction to meta-analysis. Chichester, U.K.: John Wiley & Sons; 2009.

[38] Valentine JC, Pigott TD, Rothstein HR (2010). How many studies do you need? A primer on statistical power for meta-analysis. J Educ Behav Stat.

